# FGFR1 overexpression promotes resistance to PI3K inhibitor alpelisib in luminal breast cancer cells through receptor tyrosine kinase signaling-mediated activation of the estrogen receptor

**DOI:** 10.20517/cdr.2024.181

**Published:** 2025-05-28

**Authors:** Yujie Shi, Lexia Chen, Qiong Cheng, Peijia Niu, Yahan Weng, Xiaohe Yang

**Affiliations:** ^1^Department of Pathology, People’s Hospital of Zhengzhou University, Zhengzhou 450003, Henan, China.; ^2^Department of Pathology, Fuwai Central China Cardiovascular Hospital, Zhengzhou 450003, Henan, China.; ^3^Department of Biological and Biomedical Sciences, Biomedical/Biotechnology Research Institute, North Carolina Research Campus, North Carolina Central University, Kannapolis, NC 28081, USA.; ^#^Authors contributed equally.

**Keywords:** FGFR1, alpelisib, resistance, PI3K, estrogen receptor, fulvestrant, AZD4547

## Abstract

**Aim:** Resistance to PI3K inhibitor alpelisib is an emerging challenge in breast cancer treatment. FGFR1 is frequently amplified in breast cancer. We investigated FGFR1 overexpression-mediated alpelisib resistance and its mechanism.

**Methods:** CCK-8, colony formation, and cell cycle assays assessed FGFR1 overexpression-induced alpelisib resistance in MCF-7 and T47D cells. FGFR1 siRNA knockdown validated FGFR1’s role. Akt, Erk, and ER signaling were analyzed by Western blot. Synergistic effects of alpelisib with AZD4547 and fulvestrant were evaluated using the combination index.

**Results:** FGFR1 overexpression conferred alpelisib resistance in MCF-7 and T47D cells, evidenced by increased viability, colony formation, and S-phase accumulation post alpelisib treatment. Knockdown of FGFR1 reverse alpelisib resistance in FGFR1 overexpressing MCF-7 and T47D cells. Resistance correlated with sustained activation of Akt and Erk1/2 pathways (p-Akt, p-Erk1/2, p-S6K, p-Rb) and attenuated suppression of ERα phosphorylation (S118/S167), highlighting RTK-ER crosstalk. Combining alpelisib with AZD4547 synergistically inhibited growth and suppressed both RTK signaling and ERα phosphorylation. While alpelisib-fulvestrant was effective, adding AZD4547 further enhanced inhibition, supporting triple therapy to overcome resistance.

**Conclusion:** Our findings establish FGFR1 as a key mediator of alpelisib resistance in ER+ breast cancer. Combining FGFR1 inhibitors with alpelisib-based therapies offers a viable approach for FGFR1-overexpressing tumors.

## INTRODUCTION

Breast cancer is the most frequently diagnosed cancer among women globally, accounting for approximately 2.1 million new cases annually and remaining a leading cause of cancer-related mortality, particularly in advanced stages^[1]^. Its development is driven by multifactorial processes, including genetic mutations (e.g., in *BRCA1/BRCA2* and *PIK3CA* genes) and hormonal influences such as prolonged estrogen exposure, which promote tumor initiation and progression^[1]^. Pathogenesis is further complicated by tumor heterogeneity and adaptive resistance mechanisms that reduce the efficacy of conventional therapies, posing significant treatment challenges^[2]^. Thus, effective breast cancer management remains a major challenge in oncology^[3-5]^.

Recent advances in targeted therapies offer new opportunities to improve patient outcomes^[6]^. Among these, the development and clinical application of phosphoinositide 3-kinase (PI3K) inhibitors marks a significant advancement. The PI3K signaling pathway plays a central role in the development and progression of breast cancer^[7]^. As a key target downstream of receptor tyrosine kinases, PI3K activates Akt and promotes cancer cell survival, proliferation, and invasion. Mutations and aberrant activation of PI3K are frequently implicated in tumor initiation, progression, and prognosis^[8]^. Several novel PI3K-targeted agents have received FDA approval for cancer treatment, highlighting the importance of understanding cellular responses to these therapies^[9]^.

Alpelisib is a novel selective inhibitor of the p110α catalytic subunit of PI3K and has recently been approved for treating advanced estrogen receptor-positive, HER2-negative (ER+, HER2-) breast cancer, which is mainly the luminal subtypes^[10,11]^. Its clinical use has shown promising efficacy, especially in combination with endocrine therapy^[12]^. Despite these benefits, the development of therapeutic resistance remains a significant challenge. Recent studies suggest that alterations in key regulators contribute to alpelisib resistance^[13]^. Reports indicate that resistance mechanisms may include genetic mutations, tumor microenvironment factors, and activation of alternative signaling pathways, such as secondary mutations in *PIK3CA*, PTEN loss or inactivation, and *ESR1* mutations^[13,14]^. Resistant cancer cells may also activate bypass signaling pathways, like the MAPK pathway, and undergo molecular adaptations to bypass PI3K inhibition, thereby maintaining proliferation and survival^[15,16]^. Therefore, it is critical to investigate alpelisib’s mechanisms of action and to identify factors that influence responsiveness in breast cancer cells to enhance its therapeutic potential^[11,12]^. Identifying and characterizing these resistance mechanisms is essential for developing strategies to restore sensitivity to alpelisib.

FGFR1, a receptor tyrosine kinase (RTK), is critical for normal mammary gland development and breast cancer pathogenesis^[17]^. Its activation leads to phosphorylation of downstream PI3K-Akt and MAPK-Erk1/2 pathways, which promote cancer cell proliferation and survival^[18]^. Dysregulation of FGFR1, including amplification, mutations, and overexpression, is prevalent in breast cancer, which has been associated with poor prognosis in breast cancer^[19-22]^. We previously demonstrated that FGFR1 serves as a negative prognostic marker in luminal A breast cancer^[23]^. In light of FGFR1’s pathogenic role, several therapeutic agents targeting FGFR1 have been developed for cancers with FGFR1 dysregulation^[18]^. Notably, FGFR1’s role in therapeutic resistance has garnered increasing attention^[18,24]^. Overexpression of FGFR1 has been implicated in resistance to various therapies. For instance, FGFR1 signaling enhances resistance to chemotherapeutics like cisplatin and 5-fluorouracil by activating pro-survival pathways that inhibit apoptosis^[25]^. FGFR1 signaling can also confer resistance to HER2 inhibitors by enabling transactivation of HER2 via signals from tumor-associated fibroblasts^[26]^. In non-small cell lung cancer, FGFR1 overexpression can activate bypass signaling, undermining the efficacy of EGFR inhibitors^[27]^. FGFR1 alterations have also been linked to resistance to endocrine therapies^[24]^. Both our work and that of others demonstrate that FGFR1 overexpression induces resistance to CDK4/6 inhibitors in breast cancer cells^[28-30]^. Collectively, these findings underscore the significant role of FGFR1 overexpression in therapeutic resistance, highlighting the importance of targeting FGFR1 signaling to improve treatment outcomes across various cancers. Given the potential interplay between FGFR1 and alpelisib’s target, it is crucial to investigate whether and how FGFR1 overexpression may induce alpelisib resistance in breast cancer.

In this study, we aim to investigate the effect of FGFR1 overexpression on alpelisib resistance in breast cancer cells. Using paired isogenic MCF-7 breast cancer sublines with differing FGFR1 status, we demonstrated FGFR1-induced resistance to alpelisib and characterized molecular changes in the dysregulated crosstalk between RTK and ER pathways. Additionally, we tested strategies to overcome this resistance, providing preclinical evidence for the development of combination targeting approaches to circumvent resistance and enhance therapeutic efficacy in breast cancer management.

## METHODS

### Reagents and antibodies

Alpelisib, AZD4547, and fulvestrant were ordered from LC Laboratories (Woburn, MA, USA). Primary antibodies against FGFR1 (Cat: 9740), phosphorylated Akt (Ser473) (p-Akt, Cat: 4060), phosphorylated ERK1/2 (Thr202/Tyr204) (p-ERK1/2, Cat: 9101), phosphorylated pRb (Ser807/811) (p-Rb, Cat: 8516), Rb (Cat: 9309), phosphorylated S6K (Thr389) (p-S6K, Cat: 9234), Cyclin D1 (Cat: 55506), phosphorylated ERα (Ser118 and Ser167) (p-ERα S118, Cat: 2511), phosphorylated ERα (Ser167) (p-ERα S167, Cat: 64508), ERα (Cat: 13258), Akt (Cat: 9272), ERK1/2 (Cat: 9102), and S6K (Cat: 9202), along with anti-mouse and anti-rabbit HRP-linked secondary antibodies, were purchased from Cell Signaling Technology (Danvers, MA, USA). The antibody against GAPDH (Cat: sc-47724) was procured from Santa Cruz Biotechnology (Santa Cruz, CA, USA).

### Cell culture and cell lines

The MCF-7 and T47D breast cancer cell line was obtained from the American Type Culture Collection (ATCC) (Manassas, VA, USA). Stable sublines of control and FGFR1-overexpressing MCF-7 and T47D cells, i.e., MCF-7/C *vs.* MCF-7/FGFR1; T47D/C *vs.* T47D/FGFR1 cells, were established in our laboratory as described previously^[31]^. Cells were cultured in DMEM/F-12 medium supplemented with 10% fetal bovine serum (FBS), 100 µg/mL penicillin, and 100 µg/mL streptomycin at 37 °C in a humidified atmosphere containing 5% CO_2_. Cells were passaged with 0.25% trypsin-EDTA at 80%-90% confluency.

### Survival faction assay

The survival fractions of treated cells were determined using the Cell Counting Kit-8 (CCK-8) assay. Briefly, cells were plated at a density of 1 × 10^3^ cells per well in 96-well plates. After 24 h of incubation, cells were treated with alpelisib, AZD4547, fulvestrant, or their combinations for 4 days at the concentrations specified in the results. At the endpoint, 10 μL of CCK-8 reagent was added to each well, followed by a 2-hour incubation at 37 °C. Absorbance was recorded at 450 nm using a BioTek microplate reader. Viability data were normalized to untreated controls, with each experiment performed in quintuplicate. IC_50_ values and statistical analysis were determined using GraphPad software.

### Clonogenic assays

For the clonogenic assays, 600 cells were plated in each well of 6-well plates. Following a 24-hour incubation, the cells were treated with specified concentrations of alpelisib, AZD4547, or combinations, and were cultured for 2 weeks. Colonies were fixed with 4% formaldehyde and stained with 0.5% crystal violet. Colony imaging was conducted with a Nikon SZX2-ZB10 microscope, while colony quantification was performed using ImageJ software. Data based on the results from triplicates were analyzed.

### Cell cycle analysis via flow cytometry

**Figure 1:**
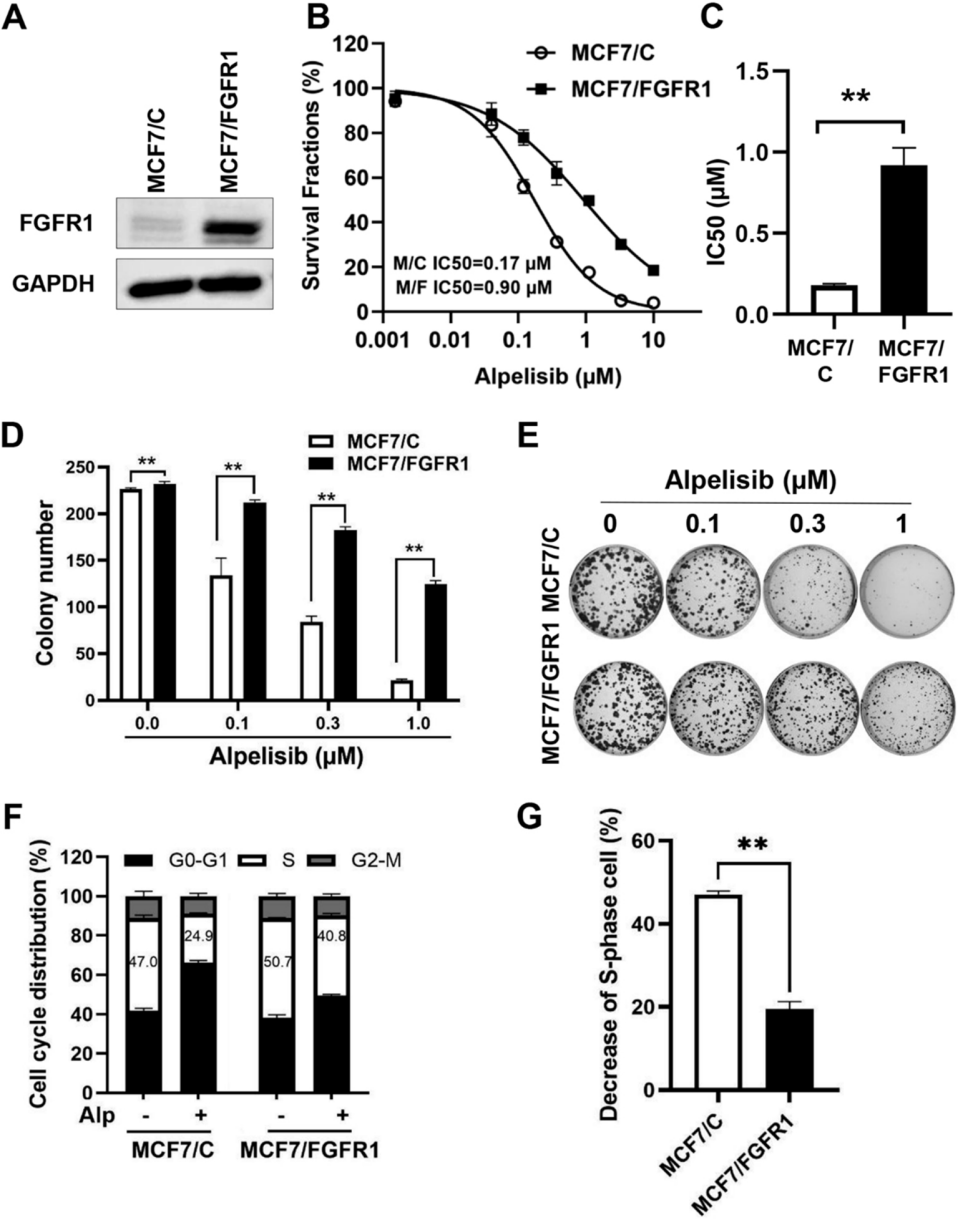
FGFR1 overexpression promotes resistance to alpelisib in MCF-7 cells. (A) Protein levels of FGFR1 in MCF7/C and MCF7/FGFR1 cells detected by Western blotting; (B) Alpelisib-induced inhibition of MCF7/C and MCF7/FGFR1 cells assessed with the CCK-8 assay. Cells were treated with alpelisib at indicated concentrations for five days, and survival fractions were determined. IC_50_ values were calculated using GraphPad Prism software; (C) IC_50_ values based on three experiments were analyzed with Welch’s *t*-test. ^**^P < 0.01; (D) Clonogenic assays of MCF7/C and MCF7/FGFR1 cells treated with alpelisib. Cells were seeded at 600 cells/well and treated with alpelisib for two weeks, followed by crystal violet staining and quantification; (E) Representative images from the clonogenic assay as described in (D); (F) Cell cycle analysis of MCF7/C and MCF7/FGFR1 cells treated with 0.3 μM alpelisib (Alp) for 24 h, followed by flow cytometry analysis in triplicate. Percentages of cells in the S phase are indicated for each sample; (G) Decrease in S-phase cells between alpelisib-treated MCF7/C and MCF7/FGFR1 cells as assessed in (F). The percentage of S-phase cells in drug-treated samples was normalized by dividing by the percentage of S-phase cells in the corresponding untreated cell line. ^**^P < 0.01.

Cells were treated with defined concentrations of alpelisib and/or AZD4547 for 24 h, followed by fixation in 70% ethanol at -20 °C overnight. After washing with PBS, the cells were stained at 37 °C for 30 min in 0.05% Triton X-100/PBS, RNase A (100 μg/mL), and propidium iodide (PI, 50 μg/mL). Cell cycle analysis was performed using a Beckman Coulter flow cytometer, and the resulting data were analyzed using ModFit software. For relative decreases in S phase cells between drug-treated MCF-7/C and MCF-7/FGFR1 cells, the percentage of S phase cells in drug-treated samples was divided by the percentage of S phase cells in the corresponding untreated cell line, which was summarized in Figure 1F.

### Drug synergy analysis

The synergistic effects of drug combinations were evaluated through CCK8 assays as in our previous reports^[29]^. The cells were treated with various drug combinations as specified in each experiment. Absorbance readings were utilized to calculate combination index (CI) values using the and CompuSyn software by Chou-Talalay *et al.*^[32]^. Based on the refined definition of CI by Chou and Talalay^[32]^, only CI values corresponding to a fractional effect (FA) greater than 0.5 were considered relevant, which were included in the plots. A CI value of less than 1.0 indicates synergy, a value of exactly 1 suggests an additive effect, and any value greater than 1 signifies antagonism.

### Western blot Analysis

Cell lysates were prepared in accordance with established protocols^[33]^. Protein concentrations were determined by the BCA Protein Assay kit (Thermo Fisher Scientific, Waltham, MA, USA). Equal amounts of protein (50 μg) were separated through SDS-PAGE and subsequently transferred to PVDF membranes. Membranes were incubated with primary antibodies overnight at 4 °C, followed by incubation with secondary antibodies for two h at room temperature. After each antibody incubation, membranes were washed four times with TBST. Detection was conducted using ECL reagents, and images were captured with a FluorChemE imaging system.

### siRNA-mediated knockdown of FGFR1

FGFR1 knockdown was performed using siRNA transfection in MCF-7/FGFR1 and T47D/FGFR1 cells. The FGFR1-targeting siRNA oligonucleotide (sequence: 5’-GCAAGATTGGCCCAGACAA-3’) and a non-targeting scrambled control siRNA were synthesized by RiboBio Co., Ltd. (Guangzhou, China). Cells at 2.5 × 10^5^/well in 6-well plates were transfected with 30 nM siRNA using Lipofectamine RNAiMAX (Thermo Fisher Scientific) according to the manufacturer’s protocol. Twenty-four h after transfection, cells were trypsinized and reseeded for subsequent proliferation and clonogenic assays.

### Statistical analysis

Data are reported as mean ± standard error. Statistical analyses comparing two groups in single-factor experiments were performed using a two-tailed Student’s *t*-test. Data from siRNA and drug treatment experiments were analyzed using a two-way ANOVA. IC_50_ values based on three independent experiments were analyzed with Welch’s *t*-test. All computations were performed using GraphPad Prism software.

## RESULTS

### FGFR1 overexpression confers resistance to alpelisib in MCF-7 breast cancer cells

To investigate the implications of FGFR1 dysregulation on the effectiveness of alpelisib, we tested the responses to alpelisib in MCF-7/C and MCF-7/FGFR1 cells, which are the control and FGFR1 overexpressing sublines established in our lab^[29,31]^. Western blot analysis confirmed the overexpression of FGFR1 in the MCF-7/FGFR1 subline, indicating a significant increase in FGFR1 protein levels compared to the control subline [Figure 1A]. When the paired cell lines were treated with alpelisib across a concentration range of 0 to 10 µM using CCK-8 assays, MCF-7/FGFR1 cells showed a markedly higher survival rate compared to the control cells under the same conditions [Figure 1B]. The IC_50_ of MCF-7/FGFR1 cells (0.90 µM ± 95% CI 0.79-1.04 µM) was over 5 times that of MCF-7/C cells (0.17 µM ± 95% CI 0.16-0.19 µM) (*P* < 0.01, Figure 1C) under the given conditions, providing further corroboration. These findings strongly indicate that FGFR1 overexpression in MCF-7 cells confers significant resistance to alpelisib.

We further examined the effects of FGFR1 overexpression on critical cellular processes such as colony formation and cell cycle progression. Colony formation assays revealed that treatment with alpelisib significantly diminished both the number and size of colonies in MCF-7/C cells. In contrast, MCF-7/FGFR1 cells retained a significantly greater ability to form colonies under identical treatment conditions [Figure 1D and E]. Flow cytometric analysis of the cell cycle revealed that alpelisib induced G0/G1 phase arrest and decreased the number of cells in the S-phase in MCF-7/C cells. This effect was markedly attenuated in MCF-7/FGFR1 cells [Figure 1F]. Since MCF-7/FGFR1 cells exhibited a higher percentage of cells in the S-phase compared to control cells prior to drug treatment, we further assessed alpelisib-mediated inhibition of cell proliferation by calculating the decrease in the S-phase population in drug-treated cells relative to each corresponding untreated cell line. As shown in Figure 1G, the reduction in the S-phase population following alpelisib treatment was significantly less pronounced in MCF-7/FGFR1 cells compared to the control cells. These findings demonstrate that FGFR1 overexpression counteracts alpelisib’s inhibitory effects on colony formation and cell cycle arrest in MCF-7 cells.

### FGFR1 overexpression sustains activation of Akt and Erk1/2 pathways in alpelisib-treated MCF-7/FGFR1 cells

**Figure 2:**
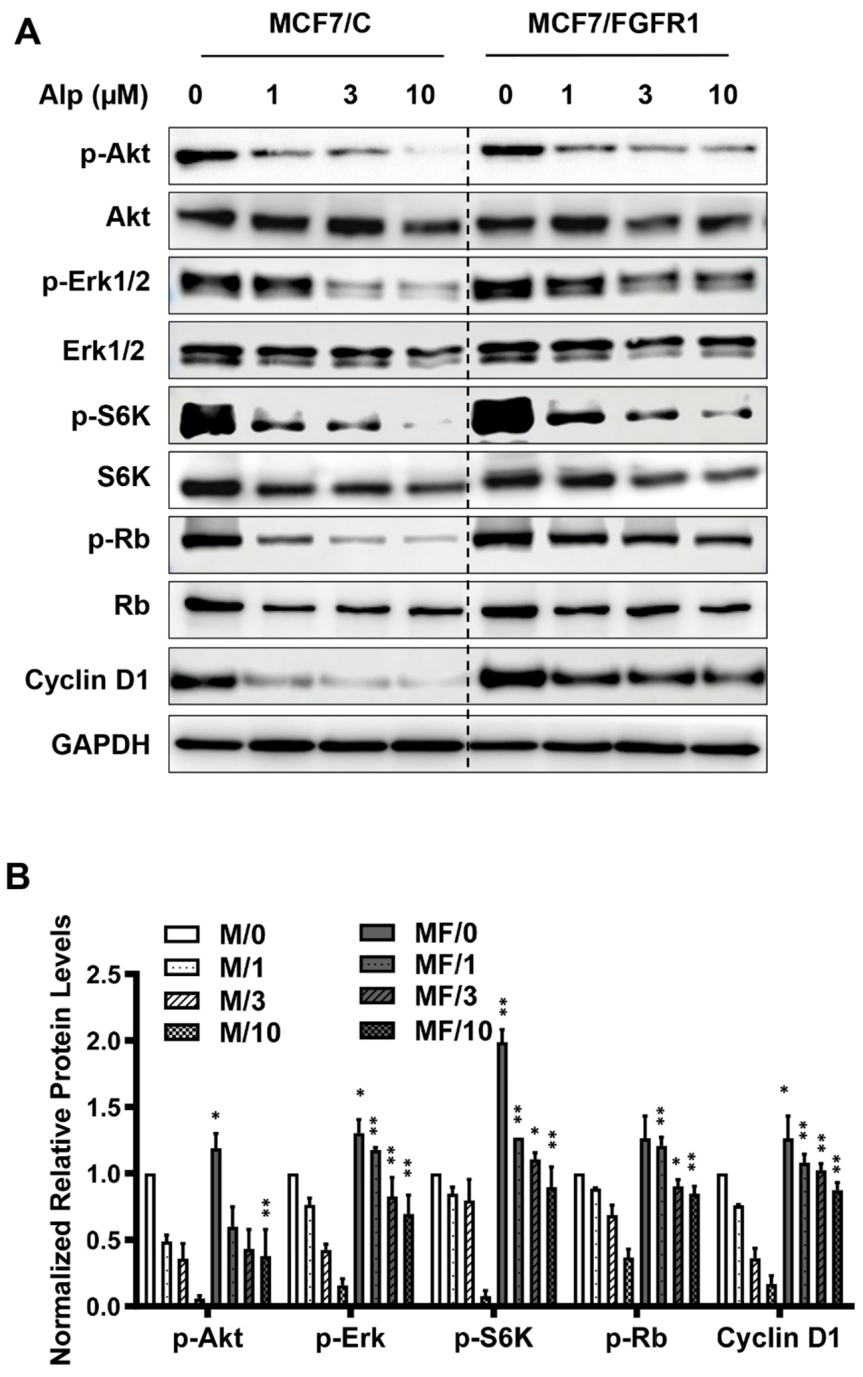
FGFR1 overexpression sustains Akt and Erk pathway activation in alpelisib-treated MCF-7/FGFR1 cells. (A) MCF7/C and MCF7/FGFR1 cells were treated with alpelisib (Alp) at the indicated concentrations for 24 h. Protein levels of p-Akt, Akt, p-Erk1/2, Erk1/2, p-S6K, S6K, p-Rb, Rb, and Cyclin D1 in each sample were detected by Western blotting, with GAPDH used as an internal control; (B) Densitometry analysis of phosphorylated/active signaling markers (p-Akt, p-Erk, p-S6K, p-Rb) and Cyclin D1 in MCF-7/C (M) and MCF-7/FGFR1 (MF) cells treated with alpelisib. Band intensities in (A) were quantified (ImageJ), normalized to loading controls (GAPDH) and total protein levels based on three repeats. ^*^P < 0.05; ^**^P < 0.01, based on comparisons of the marker between MCF-7/FGFR1 and MCF-7/C cells under the same treatment conditions.

The PI3K/Akt/mTOR/S6K pathway, the target of alpelisib, and the MAPK/Erk1/2 pathway are major targets downstream of FGFR1 signaling that promotes cell proliferation, metabolism, and survival^[34,35]^. To further explore the mechanisms underlying FGFR1-mediated resistance to alpelisib, we examined key markers in these pathways, including Akt, Erk1/2, S6K, Rb, and Cyclin D1 in alpelisib-treated MCF-7/C and MCF-7/FGFR1 cells [Figure 2A]. Alpelisib treatment significantly downregulated phosphorylated/activated (p-) Akt, Erk1/2, S6K, Rb, and the expression of Cyclin D1 in MCF-7/C cells. Conversely, densitometric analysis revealed a marked upregulation of phosphorylated (functional) forms of key markers in RTK and cell cycle signaling components in alpelisib-treated MCF-7/FGFR1 cells compared to control MCF-7/C cells [Figure 2B]. Notably, the levels of p-Akt, p-Erk, p-S6K, p-Rb, and Cyclin D1 were consistently elevated in MCF-7/FGFR1 cells across increasing doses of alpelisib. These data suggest that FGFR1 overexpression-induced activation and sustainment of the PI3K/Akt/mTOR/S6K and MAPK/Erk1/2 pathways, along with the resulting cell cycle progression, may compensate for alpelisib-mediated inhibition of these signaling pathways, contributing to drug resistance.

### FGFR1 knockdown reverses alpelisib resistance in FGFR1-overexpressing MCF-7 cells

**Figure 3:**
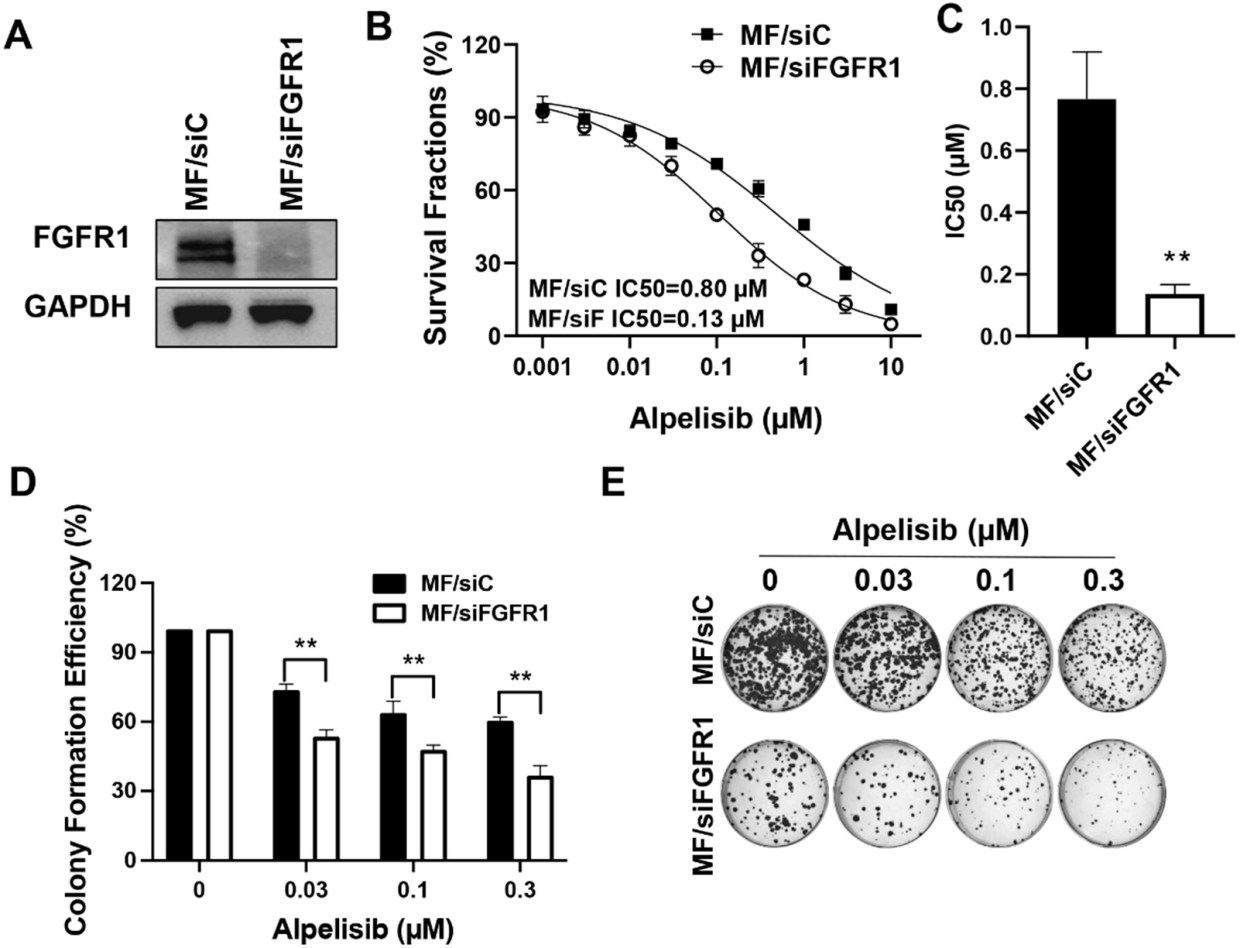
FGFR1 knockdown restores alpelisib sensitivity in FGFR1-overexpressing MCF-7 cells. (A) Immunoblot analysis of FGFR1 protein levels in MCF-7/FGFR1 (MF) cells following transient transfection with FGFR1-targeting siRNA (MF/siFGFR1) or scramble control (MF/siC); (B) Dose-response curves of alpelisib treatment in MF cells with or without FGFR1 knockdown, demonstrating a significant reduction in IC_50_ upon alpelisib treatment; (C) The statistical differences in IC_50_ values between the two groups were analyzed using Welch’s *t*-test; (D and E) Clonogenic survival assays showing colony formation efficiency of MF cells treated with indicated concentrations of alpelisib (0.03-0.3 µM) following FGFR1 knockdown. Data represent triplicate measurements analyzed by two-way ANOVA. ^**^P < 0.01.

To mechanistically validate the role of FGFR1 in mediating alpelisib resistance, we performed loss-of-function studies in MCF-7/FGFR1 cells (MF). Transient siRNA-mediated knockdown of FGFR1 effectively attenuated FGFR1 expression [Figure 3A], confirming target engagement. Importantly, FGFR1 depletion sensitized MF cells to alpelisib, as demonstrated by a significant shift in the dose-response curve and a reduction in the IC_50_ value [Figure 3B], suggesting restored PI3K pathway inhibition. Clonogenic assays provided further functional corroboration. While alpelisib alone exhibited limited efficacy against MF/C cells, its combination with FGFR1 knockdown profoundly suppressed colony formation [Figure 3C and D], indicating that FGFR1 overexpression drives therapeutic escape by sustaining proliferative signaling. These data establish FGFR1 as a critical resistance node, where its ablation reestablishes alpelisib’s anti-proliferative effects, implicating FGFR1-PI3K crosstalk as a targetable axis in resistant disease.

### The combination of the FGFR1-targeting inhibitor AZD4547 with alpelisib induces significant synergistic inhibitory effects

**Figure 4:**
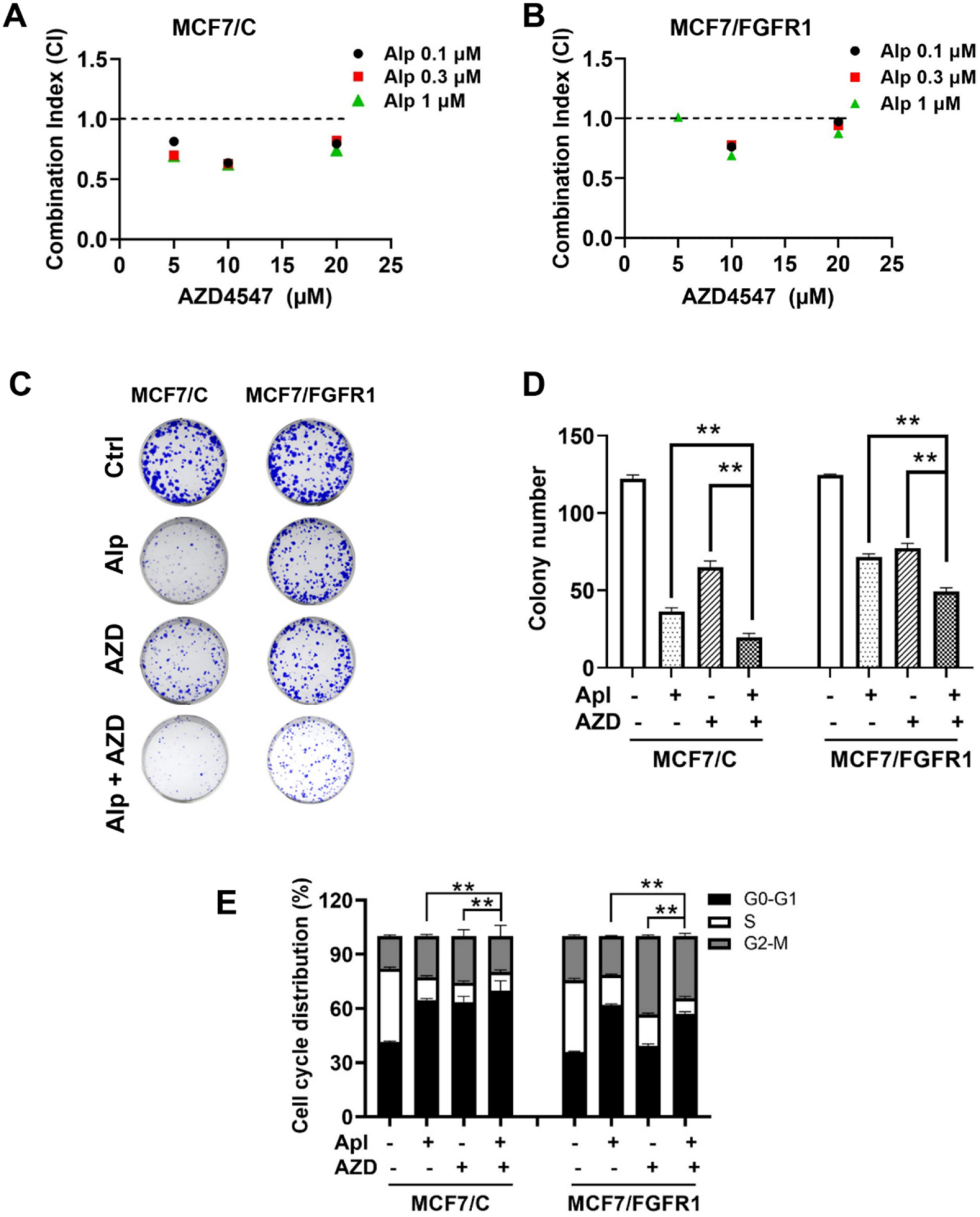
The combination of FGFR1-targeting AZD4547 with alpelisib induces significant synergistic inhibitory effects. (A) and (B) Combination index (CI) of MCF7/C (A) and MCF7/FGFR1 (B) cells treated with alpelisib (Alp) and AZD4547 in various combinations. Cells were treated with the drugs at indicated concentrations for 5 days, followed by CCK-8 assays. CI values were calculated using CompuSyn software according to the Chou-Talalay method. Only CI values corresponding to a fractional effect (FA) greater than 0.5 were presented; (C) and (D) Clonogenic assays of MCF7/C and MCF7/FGFR1 cells treated with alpelisib (Alp) or AZD4547 (AZD), alone or in combination. Cells were treated with 0.3 μM alpelisib, 3 μM AZD4547, or the combination for 2 weeks, followed by crystal violet staining; (C) Representative images of the assays; (D) Quantification of colony numbers based on triplicate experiments, displayed as a bar graph; (E) Cell cycle analysis of MCF7/C and MCF7/FGFR1 cells treated with alpelisib (Alp) or AZD4547 (AZD), alone or in combination. Cells were treated with 1 μM alpelisib, 10 μM AZD4547, or the combination for 24 h, followed by flow cytometry analysis in triplicate. ^**^P < 0.01 indicates significant differences in S-phase cell populations between the indicated groups.

Given the significant connection between FGFR1 overexpression and alpelisib resistance, we evaluated the effects of combining alpelisib with AZD4547, a selective FGFR1 inhibitor, on cancer cell inhibition. Both MCF-7/C and MCF-7/FGFR1 cells were subjected to varying concentrations of alpelisib in combination with AZD4547, as indicated in Figure 4A and B. These combination treatments produced a notable enhancement in growth inhibition assessed with CCK-8-based assays, particularly in the FGFR1-overexpressing cells. Synergy analysis using the CompuSyn program revealed that the combinations of the two agents at various concentrations resulted in a combination index (CI) of less than 1.0, indicating a synergistic interaction between the two drugs^[32]^. These results underscore the potential of targeting FGFR1 as a strategic approach to overcoming alpelisib resistance.

To further validate the synergistic effects of alpelisib and AZD4547, we evaluated colony formation efficiency and cell cycle progression in drug-treated control and FGFR1-overexpressing cells. When the cells were treated with 0.3 μM alpelisib and 3 μM AZD4547, the combination treatment substantially diminished both colony formation and the S-phase cell population in both sublines [Figures 4C-E]. While the combination is effective in enhancing cancer cell inhibition in MCF-7/FGFR1 cells, the synergistic effect observed in MCF-7/C cells suggests that the combination of alpelisib with FGFR1-targeting agents is also a meaningful approach for treating breast cancer with background levels of FGFR1. Taken together, these findings underscore the importance of supporting the therapeutic strategy of co-targeting FGFR1 to enhance the overall efficacy of alpelisib in breast cancer cells.

### Synergistic effect of alpelisib and AZD4547 combination is associated with enhanced inhibition of PI3K/Akt/mTOR/S6K and MAPK/Erk1/2 pathways

**Figure 5:**
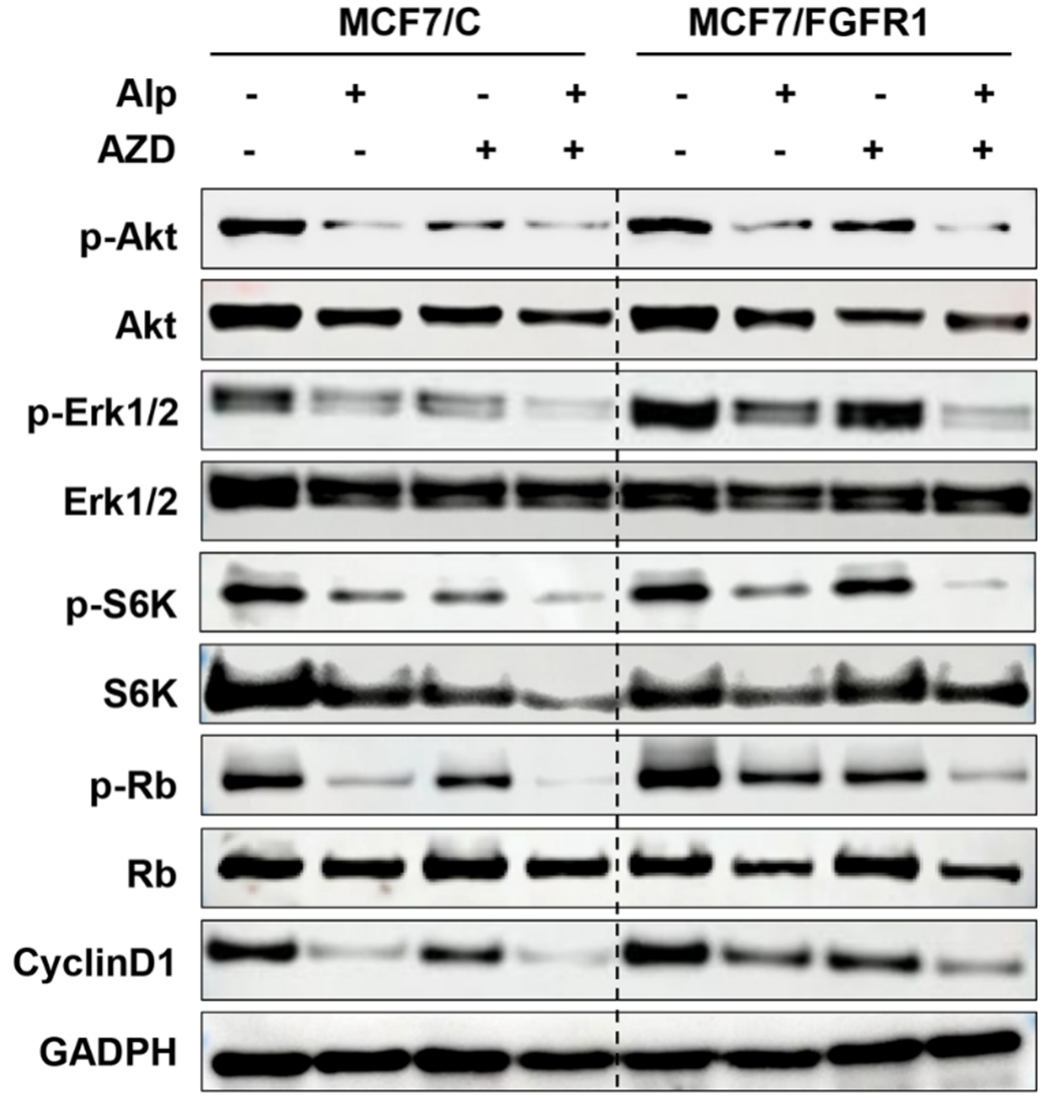
Combination of alpelisib with AZD4547 induces enhanced inhibition of the PI3K/Akt/mTOR/S6K and MAPK/Erk1/2 pathways. MCF7/C and MCF7/FGFR1 cells were treated with 2 μM alpelisib (Alp), 6 μM AZD4547 (AZD), or the combination for 24 h. Cells were then collected for Western blot analysis to assess the protein levels of p-Akt, Akt, p-Erk1/2, Erk1/2, p-S6K, S6K, p-Rb, Rb, Cyclin D1, with GAPDH as an internal control.

To understand the molecular signaling underlying alpelisib-AZD4547 combination-induced synergistic effect in MCF-7/C and MCF-7/FGFR1 cells, we examined the effect of the combination on the signaling of the PI3K/Akt/mTOR/S6K and MAPK/Erk1/2 pathways in drug-treated cells. As shown in Figure 5, the addition of AZD4547 to alpelisib treatment significantly enhanced the downregulation of phosphorylated Akt, Erk1/2, S6K, and Rb, as well as total Cyclin D1 levels, compared to treatment with each drug alone. In the context of sustained activation of these pathways in alpelisib-resistant MCF-7/FGFR1 cells, these results highlight the association between alpelisib-AZD4547 combination-induced sensitization and enhanced inhibition of the signaling in PI3K/Akt/mTOR/S6K and MAPK/Erk1/2 pathways. This supports the effectiveness of co-targeting PI3K and FGFR1 to overcome alpelisib resistance in FGFR1-overexpressing breast cancer cells.

### FGFR1-induced ERα phosphorylation/activation is a critical mediator of alpelisib resistance

**Figure 6:**
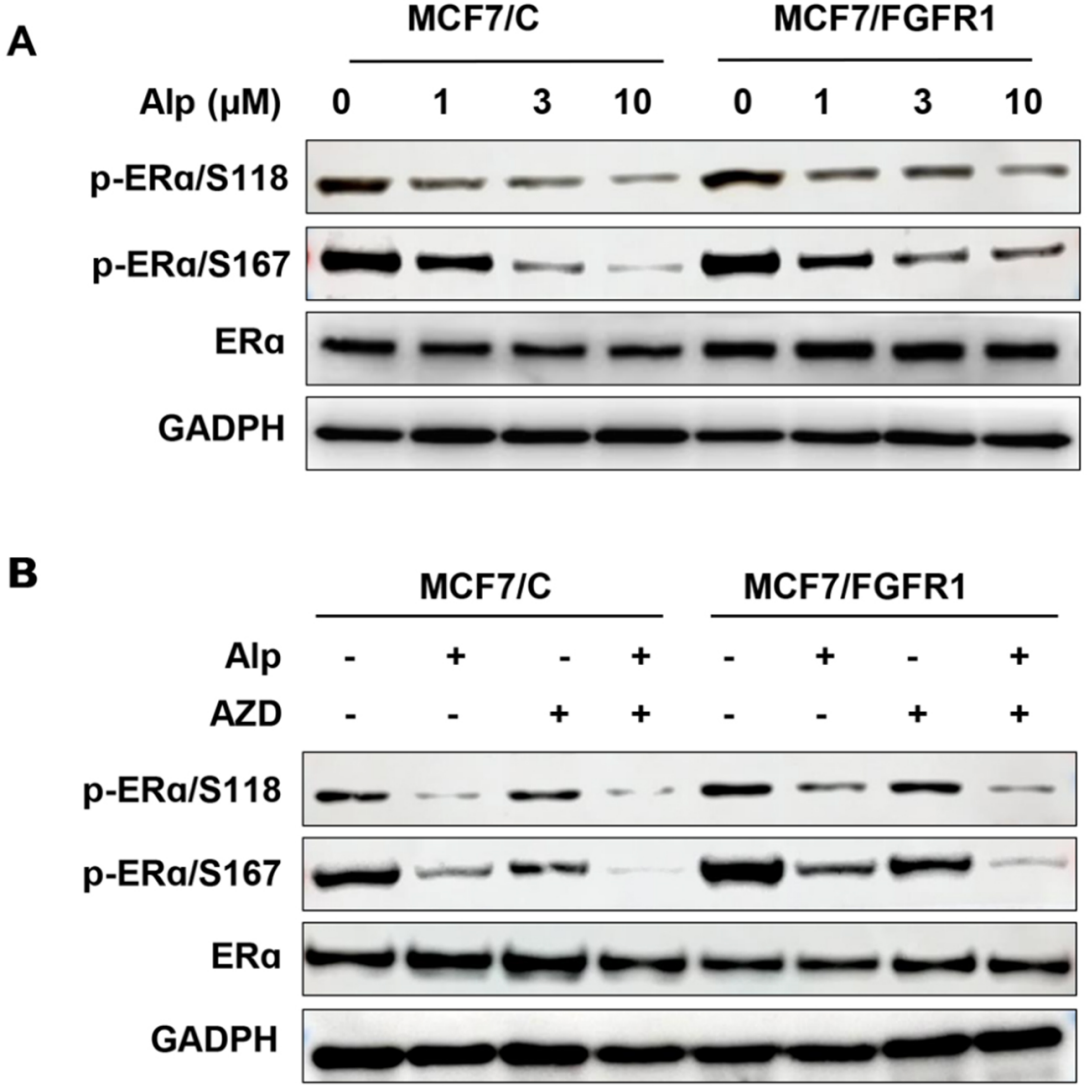
ERα phosphorylation/activation in FGFR1-driven alpelisib resistance and sensitization by combination therapy. (A) Sustained ERα phosphorylation in alpelisib (Alp)-treated MCF7/FGFR1 cells. Cells of both sublines were treated with alpelisib at the indicated concentrations for 24 h. Protein levels of p-ERα/S118, p-ERα/S167, ERα, and the internal control GAPDH were detected by Western blotting; (B) Combination of alpelisib with AZD4547 enhances inhibition of ERα phosphorylation/activation. MCF7/C and MCF7/FGFR1 cells were treated with 2 μM alpelisib (Alp), 6 μM AZD4547 (AZD), or the combination for 24 h, followed by Western blot analysis of p-ERα/S118, p-ERα/S167, ERα, and GAPDH in the indicated groups.

ER signaling plays a crucial role in breast cancer development and progression, particularly in ER+ breast cancer^[36]^. Crosstalk between RTK and ER pathways is frequently dysregulated in breast cancer oncogenesis and therapeutic resistance^[37]^. To investigate the role of ER signaling in FGFR1-induced alpelisib resistance, we analyzed total and phosphorylated ERα levels in alpelisib-treated MCF-7/C and MCF-7/FGFR1 cells [Figure 6A]. The results showed that alpelisib induced significant downregulation of phosphorylated ERα at both S118 and S167 sites in control MCF-7 cells. However, in MCF-7/FGFR1 cells, alpelisib-induced downregulation of pERα/S118 and pERα/S167 was significantly attenuated, which was accompanied by elevated total ERα levels, suggesting that FGFR1 overexpression-induced ERα expression and phosphorylation may contribute to FGFR1-induced alpelisib resistance. To demonstrate the impact of FGFR1 function on ERα activation in context with the combination treatment, we tested ERα expression and phosphorylation in response to alpelisib in the absence and presence of FGFR1-targeting AZD4547 in the paired cell lines. As shown in Figure 6B, the co-treatment with AZD4547 induced enhanced inhibition of both pERα/S118 and pERα/S167 in both cell lines, particularly MCF-7/FGFR1 cells, indicating that FGFR1 activation leads to enhanced ERα phosphorylation, and co-targeting PI3K and FGFR1 is effective in blocking ER signaling in alpelisib-treated breast cancer cells. Notably, pERα/S118 and pERα/S167 are substrates of Erk and Akt pathways, respectively^[37]^. In context with FGFR1 overexpression-mediated activation of Akt and Erk1/2 [Figures 2 and 5], these data demonstrate that FGFR1-induced ERα phosphorylation and crosstalk between RTK-ER pathways are key mechanisms of alpelisib resistance in breast cancer cells.

### FGFR1-targeting AZD4547 enhances combined therapy with alpelisib and fulvestrant

**Figure 7:**
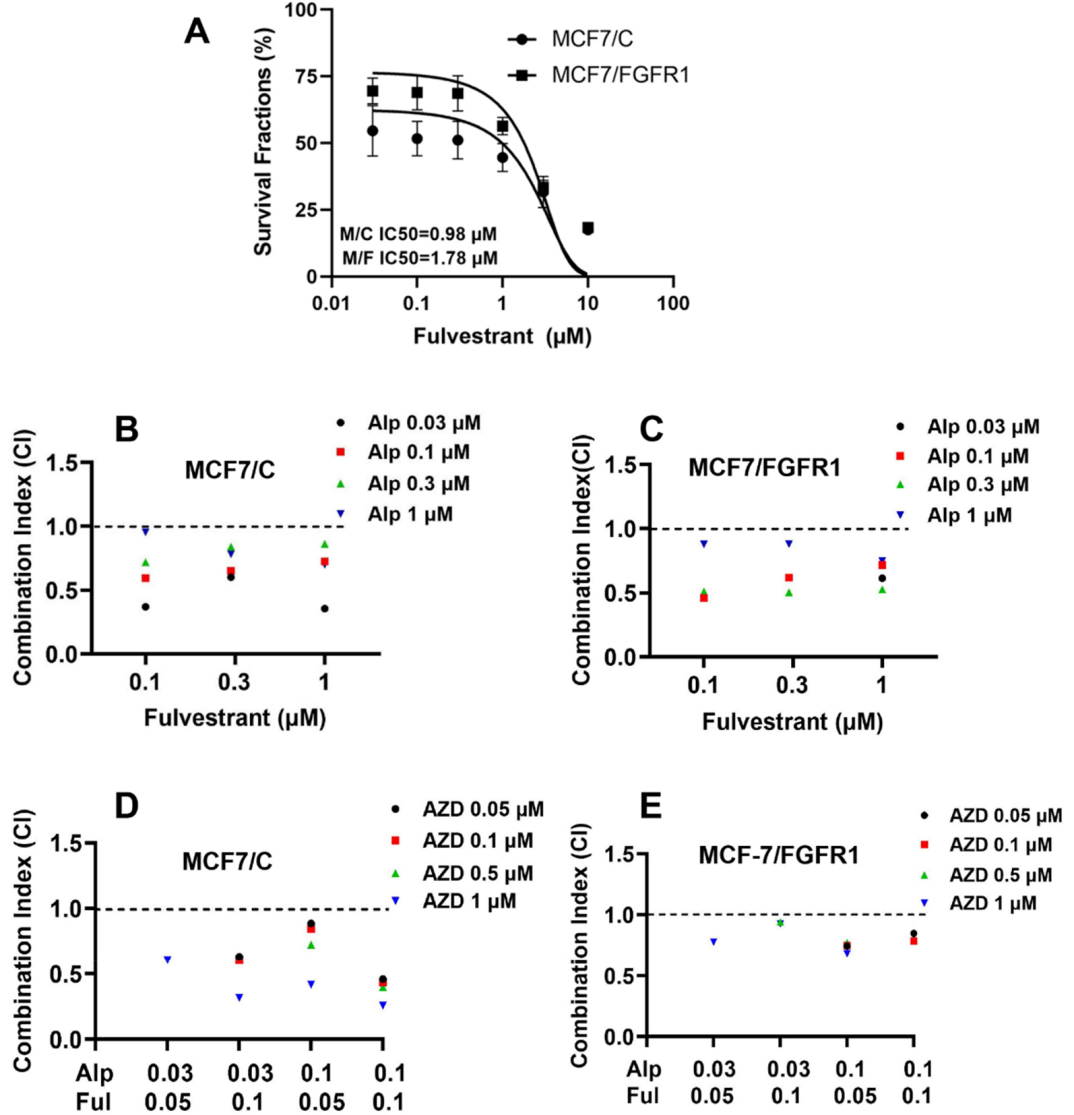
The combination of alpelisib with fulvestrant induces a synergistic effect that is further enhanced by the addition of AZD4547. (A) Fulvestrant-induced inhibition of MCF7/C and MCF7/FGFR1 cells. Cells were treated with fulvestrant at the indicated concentrations for 5 days, followed by CCK-8 assays. IC_50_ values were analyzed with GraphPad; (B and C) Synergistic effect of alpelisib with fulvestrant in MCF7/C (B) and MCF7/FGFR1 (C) cells. Cells were treated with alpelisib (Alp) and fulvestrant at the indicated concentrations for 5 days, followed by CCK-8 assays. CI values were calculated using CompuSyn software according to the Chou-Talalay method. Only CI values corresponding to a fractional effect (FA) greater than 0.5 were presented; (D and E) Addition of AZD4547 (AZD) further enhances the alpelisib-fulvestrant (Alp-Ful) combination in four different treatment regimens. MCF7/C (D) and MCF7/FGFR1 (E) cells were treated with AZD4547 and four different combinations of alpelisib and fulvestrant at the indicated concentrations for 5 days, followed by CCK-8 assays. CI values were calculated as above.

The data above indicate that ER signaling plays a critical role in alpelisib resistance mediated by FGFR1-induced ER-RTK crosstalk. Since alpelisib is primarily used for breast cancer of luminal subtypes^[38]^, a common clinical regimen involves combining alpelisib with ER-targeting therapeutics, such as fulvestrant, which improves treatment outcomes^[12]^. Given the increased activation of ER signaling in alpelisib-treated MCF-7/FGFR1 cells, we next tested whether adding the FGFR1-targeting agent AZD4547 could enhance the efficacy of the alpelisib-fulvestrant regimen. To begin, we assessed the relative response of the paired cell lines to fulvestrant to guide the design of the tri-agent combination [Figure 7A]. The IC_50_ values for MCF-7/C and MCF-7/FGFR1 cells with fulvestrant treatment were 0.98 and 1.78 μM, respectively, indicating that FGFR1 overexpression confers resistance to fulvestrant as a single agent.

We then tested the combination of alpelisib (0.03-1 μM) with fulvestrant (0.1-1 μM) [Figure 7B and C]. The results showed a strong synergistic effect in both cell lines, supporting the use of the alpelisib-fulvestrant combination for enhanced cancer cell inhibition. Based on these findings, we evaluated whether adding an FGFR1-targeting agent could further enhance alpelisib-fulvestrant therapy. We tested the effect of AZD4547 (0.05-1 μM) on MCF-7/C and MCF-7/FGFR1 cells treated with four alpelisib-fulvestrant combinations (A 0.03 + F 0.05; A 0.03 + F 0.1; A 0.1 + F 0.05; A 0.1 + F 0.1 μM) [Figure 7D and E]. The results demonstrated that adding AZD4547 induced a further synergistic effect with the alpelisib-fulvestrant combination, as indicated by a CI value < 1.0. Together, these data suggest that adding FGFR1-targeting agents to the current alpelisib-fulvestrant regimen enhances cancer inhibition.

### FGFR1 overexpression in T47D cells confers resistance to alpelisib

**Figure 8:**
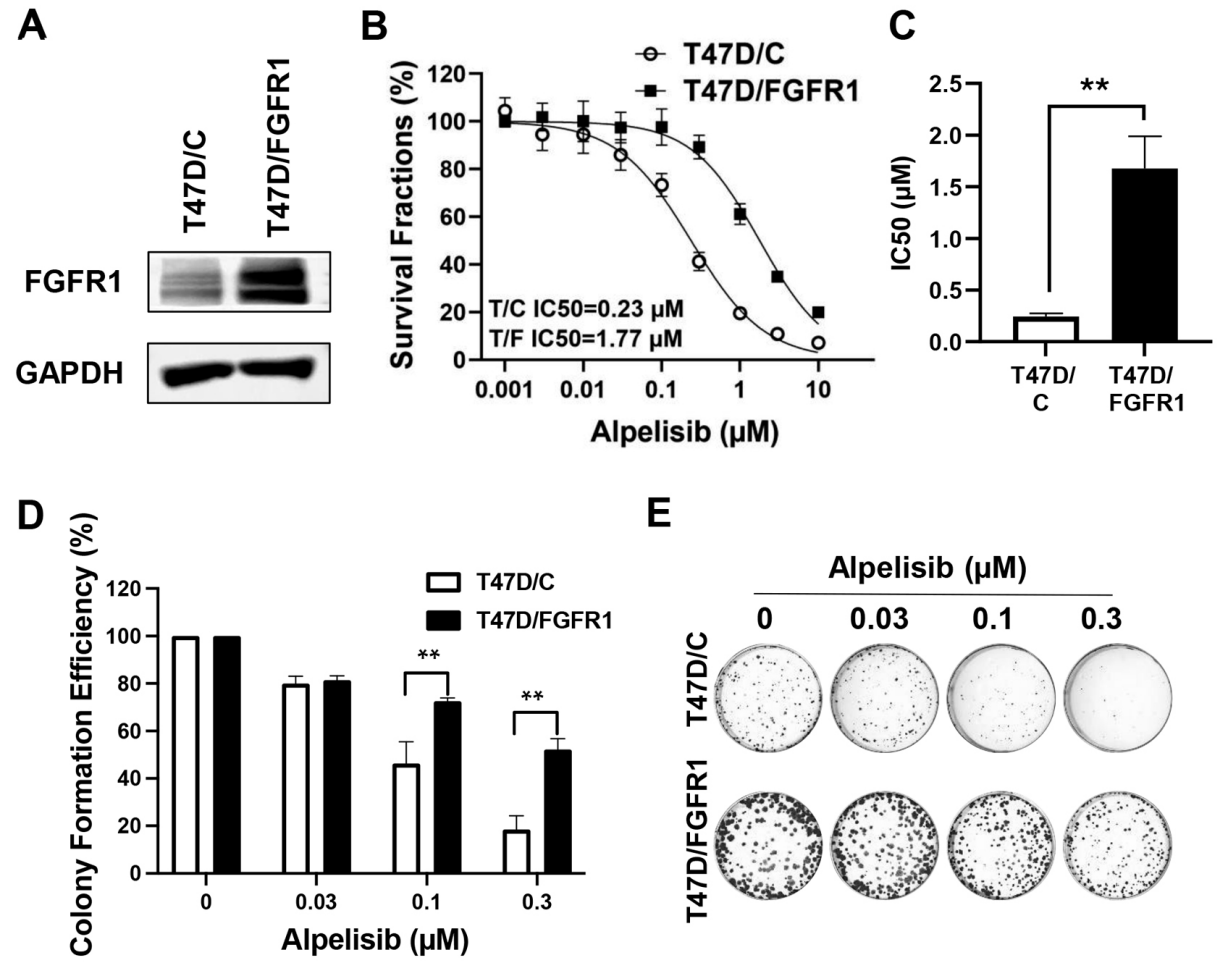
FGFR1 overexpression confers alpelisib resistance in T47D cells. (A) Immunoblot analysis of FGFR1 overexpression in T47D/FGFR1 and control T47D/C cells; (B) Dose-response curves of T47D/C and T47D/FGFR1 cells measured with CCK-8 assays; (C) Quantification of IC_50_ values derived from the dose-response assays in panel B. Statistical differences in IC_50_ values between the two groups were analyzed using Welch’s *t*-test. ^**^P < 0.01; (D and E) Clonogenic survival assays of T47D/C (control) and T47D/FGFR1 cells treated with indicated concentrations of alpelisib, performed using the same methodology as in Figure 1D and E. ^**^P < 0.01.

To validate our findings from MCF-7 cells, we extended our investigation to T47D breast cancer cells, a well-characterized luminal breast cancer cell line, using the stable T47D/C and T47D/FGFR1 cell lines previously generated in our laboratory. Immunoblot analysis confirmed robust overexpression of FGFR1 in T47D/FGFR1 cells compared to vector controls [Figure 8A]. Dose-response assays demonstrated that FGFR1 overexpression significantly increased resistance to alpelisib, as indicated by a markedly higher IC_50_ in T47D/FGFR1 cells [Figure 8B]. Clonogenic survival assays further corroborated these findings. While T47D/C cells exhibited a dose-dependent inhibition of colony formation, T47D/FGFR1 cells maintained significantly higher colony-forming efficiency at the tested doses [Figure 8C and D]. These results in a second luminal breast cancer model confirm FGFR1s as a conserved mediator of alpelisib resistance. The consistency of these findings strengthens the potential clinical relevance of FGFR1-targeted strategies to overcome resistance to PI3K inhibitors.

### FGFR1 knockdown restores alpelisib sensitivity in T47D/FGFR1 cells

**Figure 9:**
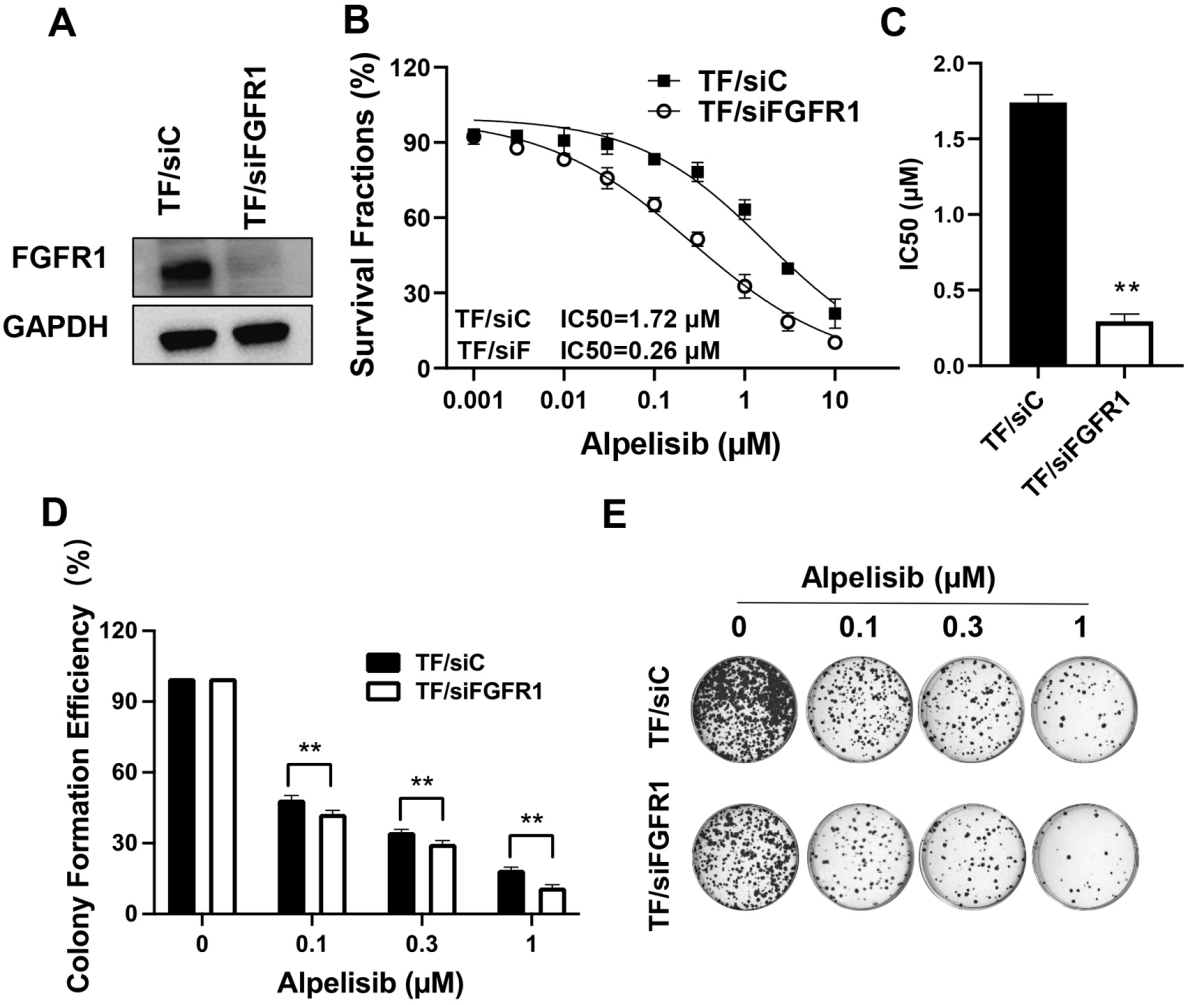
FGFR1 knockdown reverses alpelisib resistance in T47D/FGFR1 cells. (A) Immunoblot analysis of FGFR1 protein levels in T47D/FGFR1 (TF) cells following transfection with FGFR1-targeting siRNA (TF/siFGFR1) or scramble control (TF/siC); (B) CCK-8 measurement of the dose-response curves of TF/siC and TF/siFGFR1 cells followed by IC_50_ calculation; (C) Comparison of the IC_50_ values between the two groups analyzed with Welch’s *t*-test; (D and E) Clonogenic assays of TF/siC and TF/siFGFR1 cells. Twenty-four h after siRNA transfection, the cells were reseeded and treated with alpelisib at indicated concentrations. Quantified colony formation efficiencies were analyzed with a two-way ANOVA test. ^**^P < 0.01.

To confirm the functional role of FGFR1 in mediating alpelisib resistance across luminal breast cancer models, we performed siRNA-mediated FGFR1 knockdown in T47D/FGFR1-overexpressing cells (TF). Efficient FGFR1 suppression with GFR1-specific siRNA [Figure 9A] sensitized cells to alpelisib, as evidenced by a significant decrease in IC_50_ (1.72 μM *vs.* 0.26 μM, *P* < 0.01) [Figure 9B]. Clonogenic assays demonstrated markedly enhanced drug response, with FGFR1-depleted cells showing enhanced colony formation inhibition, as compared to the control cells [Figure 9C and D]. These results recapitulate our findings in MCF-7 cells and support FGFR1 as a mediator of alpelisib resistance in luminal breast cancer cells.

### Targeting FGFR1 with AZD4547 enhances alpelisib sensitivity and synergizes with alpelisib and fulvestrant in T47D cells

**Figure 10:**
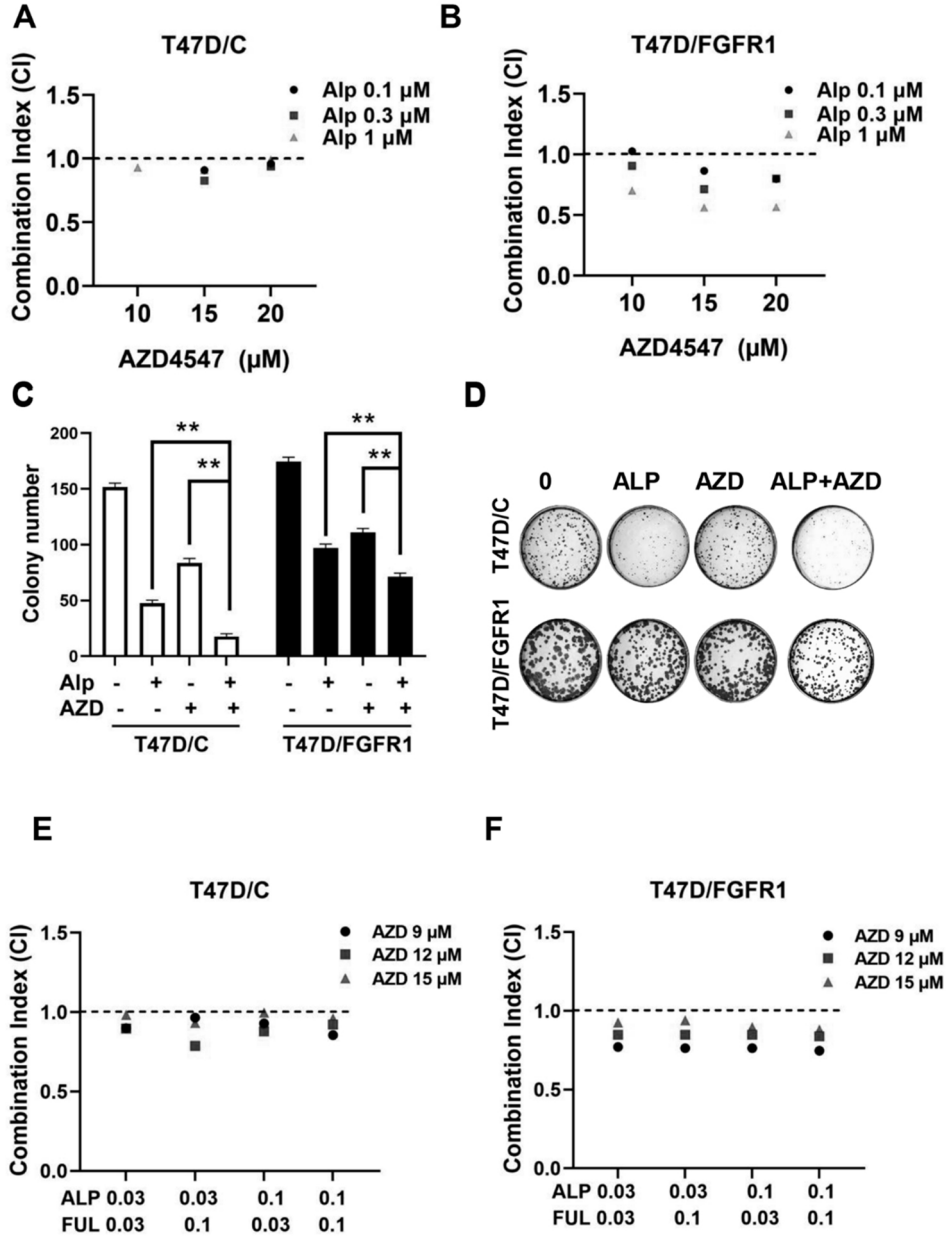
FGFR1 inhibition enhances alpelisib efficacy and synergizes with alpelisib + fluvastatin in T47D cells. (A and B) Synergistic interaction between alpelisib (ALP) and AZD4547 (AZD) in T47D/C and T47D/FGFR1 cells. Combination index (CI) values were calculated using the Chou-Talalay method (as described in Figure 4), with CI < 1 indicating synergism; (C and D) Effect of the alpelisib and AZD4547 combination on colony formation in T47D/C and T47D/FGFR1 cells. The combination treatment led to enhanced inhibition of colony formation; (E and F) Synergy analysis of AZD4547 in combination with alpelisib and fluvastatin. The triple combination (ALP + Fluvastatin + AZD) significantly suppressed cell proliferation, particularly in T47D/FGFR1 cells.

To support our findings from MCF-7 cells, we evaluated the impact of the FGFR inhibitor AZD4547 on alpelisib sensitivity in T47D control (T47D/C) and FGFR1-overexpressing (T47D/FGFR1) cells. Combination index (CI) analysis revealed a strong synergy between AZD4547 and alpelisib (CI < 1), with enhanced effects in T47D/FGFR1 cells [Figure 10A and B]. Clonogenic assays further demonstrated that the combination significantly suppressed colony formation in both cell lines [Figure 10C and D]. To assess clinical relevance, we tested AZD4547 in combination with alpelisib and fulvestrant, a standard endocrine therapy. Notably, AZD4547 potentiated the efficacy of the alpelisib-fulvestrant combination in both T47D/C and T47D/FGFR1 cells [Figure 10E and F]. These results validate FGFR1 as a therapeutic target to overcome PI3K inhibitor resistance and support its inclusion in rational drug combinations for luminal breast cancer.

## DISCUSSION

In this study, we investigated the effect of FGFR1 overexpression on alpelisib resistance and its underlying mechanisms in MCF-7 and T47D breast cancer cells, two luminal subtype cell lines. Our findings show that FGFR1 overexpression reduces the efficacy of alpelisib and sustains cellular proliferation and survival by activating compensatory signaling in the Akt, Erk1/2, and ER pathways. These observations, along with the synergistic effect of the alpelisib-AZD4547 combination, provide crucial insights into the molecular basis of alpelisib resistance and highlight potential strategies to overcome it by co-targeting PI3K and FGFR1 signaling in FGFR1-overexpressing breast cancer cells.

Alpelisib has shown significant clinical efficacy, particularly in PI3KCA-mutated, HR+, HER2-negative luminal breast cancer^[39]^. However, intrinsic and acquired resistance poses a significant challenge to its potential in breast cancer treatment^[16]^. Identification of potential determinants or markers for alpelisib responsiveness/resistance is of great significance. Results from this study support the role of FGFR1 overexpression or activation in alpelisib resistance in breast cancer treatment.

Our findings revealed that MCF-7 and T47D cells with FGFR1 overexpression exhibited higher survival rates and increased colony formation under alpelisib treatment compared to control cells [Figures 1 and 8]. Conversely, silencing FGFR1 with FGFR1 targeting siRNA significantly restored sensitivity to alpelisib in both MCF-7/FGFR1 and T47D/FGFR1 cell lines [Figures 3 and 9]. These findings complement the overexpression model results and confirm that FGFR1 is both necessary and sufficient to confer resistance to PI3K inhibition in luminal breast cancer cells. These data were further supported by enhanced activation of key signaling molecules downstream of RTK pathways, including Akt, Erk1/2, S6K, and Cyclin D1 [Figure 2]. FGFR1 overexpression led to sustained activation of these pathways, mitigating the impact of alpelisib on cell proliferation and survival. This suggests that FGFR1-induced alpelisib resistance is mediated by signaling that not only compensates for PI3K inhibition but also drives key cellular processes essential for breast cancer progression. Moreover, we observed enhanced phosphorylation/activation of ERα in FGFR1-overexpressing cells. Previous studies have shown that ERα phosphorylation at S118 and S167 is regulated by Akt and Erk signaling^[37]^, both of which were elevated in our FGFR1-overexpressing cells. These data highlight the role of RTK-ER signaling crosstalk in mediating resistance and suggest ERα may serve as a key downstream effector of FGFR1, contributing to alpelisib resistance by sustaining estrogen receptor signaling in the presence of PI3K inhibition.

In Figures 2 and 5, we observed that alpelisib, a selective PI3Kα inhibitor, not only suppresses PI3K-Akt signaling but also exerts significant inhibitory effects on the MAPK/Erk1/2 pathway. This concurrent inhibition highlights the extensive crosstalk between these pathways, as both are activated by shared upstream mediators such as RAS and RTKs, so blockade of PI3Kα by alpelisib can indirectly reduce MAPK pathway activation via a reduction in signal transduction from these nodes^[10,40]^. In addition, alpelisib-induced disruption of feedback regulation, including impaired mTORC1-mediated signaling, and diminished ER-dependent growth factor expression further attenuate Erk1/2 activation^[41,42]^. This dual pathway inhibition has been substantiated in preclinical models, where it is shown to enhance the anti-proliferative efficacy of alpelisib by simultaneously impairing multiple pro-survival and mitogenic signaling networks in cancer cells^[10,43,44]^.

These findings are clinically significant, as FGFR1 dysregulation is common in ER+ breast cancer^[23,45]^. FGFR1 alterations, including overexpression, amplification, and functional activation, play a crucial role in breast cancer pathogenesis, particularly in HR+ subtypes^[20]^. FGFR1 amplifications are detected in 7.5% to 17% of all breast cancer cases, with rates rising to 16% to 27% in aggressive HR+ luminal-B tumors^[46]^. FGFR1 is the founding member of the FGFR family, which also includes FGFR2, FGFR3, and FGFR4^[47]^. Alterations in other FGFR family members also contribute to breast cancer pathogenesis through shared signaling mechanisms that promote tumor growth, survival, and metastasis, although each family member has distinct functions^[17]^. The functional consequences of these alterations include aberrant RTK signaling activation^[17]^. Therefore, our findings not only apply to FGFR1 overexpression but also to other FGFR1 alterations and FGFR family members, which warrants further investigation.

Targeting FGFR1 alongside PI3K inhibition with alpelisib reveals a strong synergistic effect, as evidenced by decreased cell viability, reduced colony formation, and suppression of S-phase entry [Figure 4]. The combination therapy significantly enhances the inhibition of critical signaling markers (e.g., p-Akt, p-Erk1/2, and p-S6K), highlighting the role of FGFR1 in sustaining pathway activity and mitigating drug efficacy in FGFR1-overexpressing cells [Figure 5]. The calculated combination index (CI) further confirms the synergy between alpelisib and AZD4547, supporting a co-targeting approach to overcome the limitations of single-agent alpelisib in FGFR1-overexpressing breast cancers. These findings are in line with prior reports where FGFR1 inhibitors restored sensitivity to the resistant cells treated with other targeted therapeutics, such as CDK4/6 inhibitors^[28]^.

Our data implicate FGFR1-mediated ERα phosphorylation as a key player in alpelisib resistance. FGFR1 overexpression attenuates alpelisib-induced downregulation of pERα/S118 and pERα/S167 in MCF-7 cells, suggesting that FGFR1 not only activates RTK signaling but also reinforces ERα activity under PI3K inhibition [Figure 6]. This is consistent with the role of RTK-ER crosstalk in breast cancer oncogenesis^[48]^. The crosstalk involves activation of RTK downstream pathways, such as Akt and Erk, which phosphorylate and activate ERα. In turn, ER activation induces the expression of FGF-like growth factors, creating a feedback loop that amplifies growth signaling^[49]^. This mechanism aligns with studies showing that ER+ breast cancers develop drug resistance through enhanced ER signaling and RTK-ERα crosstalk, leading to persistent proliferation and survival^[24,30,37]^. The reduction in ERα phosphorylation upon AZD4547 addition supports the hypothesis that FGFR1-mediated ERα activation is essential for alpelisib resistance, offering a mechanistic basis for incorporating FGFR1 inhibitors to diminish this pathway.

Results from our study support the clinical implications and therapeutic potential of triple therapy. The addition of AZD4547 to the alpelisib-fulvestrant regimen notably strengthens therapeutic efficacy, resulting in profound synergy against FGFR1-overexpressing cells [Figures 7 and 10]. This triple therapy mitigates both RTK and ER pathways, addressing the crosstalk that underpins resistance. Given that the alpelisib-fulvestrant combination is a clinical standard for ER+/HER2- breast cancer^[50]^, our results suggest that patients with high FGFR1 expression might benefit from this modified regimen, thereby enhancing clinical outcomes in resistant cases. Future clinical trials should explore the safety and efficacy of this approach in FGFR1-amplified or -overexpressing patient subsets to validate these preclinical findings. Moreover, advances in genetic diagnostics, such as those outlined by Mela *et al.*^[51]^, highlight the potential of biomarker-driven strategies to identify FGFR1-overexpressing tumors and tailor combination therapies.

Although our *in vitro* findings provide strong evidence that FGFR1 overexpression promotes resistance to alpelisib through sustained PI3K/Akt and MAPK/Erk1/2 signaling and enhanced ER transcriptional activity, we acknowledge that these results have not yet been validated *in vivo*. *In vivo* studies are essential to confirm the physiological relevance and therapeutic potential of the proposed resistance mechanisms and combination strategies. We are currently developing xenograft models to evaluate FGFR1 overexpression-associated resistance to alpelisib and validate the therapeutic synergy observed *in vitro*.

In summary, our findings establish FGFR1 as a key driver of alpelisib resistance in ER+ breast cancer by sustaining PI3K/Akt and MAPK/Erk1/2 signaling. As highlighted in the graphic abstract, in FGFR1-overexpressing cells, persistent RTK-ER crosstalk, evidenced by sustained ERα phosphorylation at S118/S167 and upregulation of ER target genes MYC, FGF, and CCND1, bypasses PI3K inhibition, promoting proliferation and survival. Notably, co-targeting FGFR1 with AZD4547 disrupts this resistance by blocking Akt and Erk1/2 activation, while fulvestrant eliminates compensatory ER signaling. The synergy of triple therapy (alpelisib/AZD4547/fulvestrant) stems from concurrent inhibition of PI3K (alpelisib), FGFR1-driven bypass pathways (AZD4547), and ER-dependent survival programs (fulvestrant). This multi-targeted strategy effectively overcomes signaling redundancy in FGFR1-high tumors, offering a promising precision therapy approach.

## DECLARATIONS

### Availability of data and materials

The raw data supporting the conclusions of this article will be made available by the corresponding authors, without undue reservation.

### Copyright

© The Author(s) 2025.
